# Metabolic syndrome among adults with type 2 diabetes in a Saudi teaching hospital: A comparative prevalence study using WHO and ATP III definitions

**DOI:** 10.12669/pjms.35.4.199

**Published:** 2019

**Authors:** Ranya A. Ghamri, Sultan H. Alamri

**Affiliations:** 1 Ranya A. Ghamri MBBS, SBFM. Department of Family Medicine, College of Medicine, King Abdulaziz University, Jeddah, Kingdom of Saudi Arabia; 2 Sultan H. Alamri, MBBS, SBFM. Department of Family Medicine, College of Medicine, King Abdulaziz University, Jeddah, Kingdom of Saudi Arabia

**Keywords:** Diabetes, Metabolic syndrome, Insulin resistance, Hypertension, Dyslipidemia, Obesity

## Abstract

**Objective:**

Metabolic syndrome (MetS) has become a global health concern and is a reliable predictor of long-term adverse health outcomes. This study aimed to determine the prevalence of MetS and its components in a group of Saudi adults with type 2 diabetes using the World Health Organization (WHO) and Adult Treatment Panel (ATP) III definitions, and to examine agreement between both definitions.

**Methods:**

This cross-sectional study included adults with type 2 diabetes who were followed up at the family medicine and endocrinology clinics of King Abdulaziz University Hospital (KAUH) from January to March 2018. An interview-administered questionnaire was designed to collect demographic data, anthropometric measurements, and medical history. We used the 1999 WHO and 2001 ATP III definitions for diagnosing MetS.

**Results:**

The study included 155 diabetes patients. The overall prevalence of MetS components (three of more components) among patients was 80% according to the WHO criteria and 85.8% according to the ATP III criteria. The kappa statistics demonstrated good agreement between both definitions (κ = 0.751, p < 0.001). The sensitivity and specificity of diagnosing MetS using the WHO versus ATP III criteria were 92.5% and 95.5%, respectively. There was weak positive association between the number of MetS components and the number of diabetic complications.

**Conclusions:**

MetS was highly prevalent among Saudi adults with type 2 diabetes regardless of the diagnostic criteria. It is, therefore, imperative that clinicians identify MetS in this patient population and educate them on the importance of adherence to treatment and therapeutic lifestyle changes.

## INTRODUCTION

Metabolic syndrome (MetS) is a clustering of four major cardiovascular risk factors, namely, atherogenic dyslipidemia, abdominal obesity, hyperglycemia (insulin resistance), and hypertension.^1^-^3^ MetS has become a global health concern and is a reliable predictor of long-term adverse health outcomes.^4^

According to the International Diabetes Federation (IDF), MetS occurs in approximately 25% of the world’s population. However, this prevalence estimate has wide variations due to differences in population ethnicity, age, and sex.^5^

Several studies have been conducted in many countries to assess MetS prevalence in people with T2DM. In a cross-sectional study in Ghana in 2015, Nsiah et al. reported that 58% of the study population (T2DM patients) had MetS. The percentage was higher in females (77.01%).^2^ In 2012, Kengne et al. reported MetS prevalence rates of 71.7% (IDF) and 60.4% (National Cholesterol Education Program - Adult Treatment Panel III [NCEP ATP III]) among T2DM patients in Sub-Saharan Africa.^6^ In 2013, a study by Yadav et al. in India also evaluated MetS prevalence in T2DM patients. The study reported prevalence rates of 57.7%, 45.8%, and 28% according to the IDF, NCEP ATP III, and World Health Organization (WHO) definitions, respectively.^7^

This present study aimed to determine the prevalence of MetS and its components among Saudi adults with T2DM using the WHO and ATP III definitions. The agreement between both definitions was also examined.

## METHODS

This cross-sectional study spanned three months (January-March 2018), and was conducted on a convenient sample of 155 adults with T2DM who were followed up at the family medicine and endocrinology clinics of King Abdulaziz University Hospital (KAUH).

All included patients were evaluated for presence of MetS according to the WHO and ATP III criteria. An interview-administered questionnaire was developed to collect demographic data (age and sex), anthropometric measurements (weight, height, waist circumference, and body mass index [BMI]), and medical history (diabetic complications such as retinopathy, neuropathy, and ischemic heart disease [IHD]). The following MetS components were documented: triglyceride, high-density lipoprotein (HDL), and fasting blood sugar levels. We also checked whether the patients were on any medication for hypertension or dyslipidemia.

### Definitions used

Different health organizations have proposed various definitions and diagnostic criteria for MetS using different medical terminologies.

In 1998, WHO first proposed the definition of MetS. According to the WHO criteria, the absolute requirement for diagnosing MetS is insulin resistance (impaired glucose intolerance, impaired fasting glucose, T2DM, or other evidence of insulin resistance).^8^ The WHO criteria state that, along with insulin resistance, two or more of four components should be present in an individual when diagnosing MetS.^8^-^10^ These components are central obesity (waist/hip ratio, >0.9 [male] and >0.85 [female]; and/or BMI, >30 kg/m^2^), hypertension (raised arterial pressure, ≥140 mmHg), dyslipidemia (raised plasma triglycerides, ≥150 mg/dL; and/or low HDL-C, <35 mg/dL [male] and <39 mg/dL [female]), and microalbuminuria (urinary albumin excretion rate, ≥20 μgm/min; or albumin/creatinine ratio, ≥30 mg/g).

In 2005, the NCEP ATP III presented a revised definition for MetS, which states that a MetS diagnosis is confirmed if three or more of the criteria are present in an individual.^8^,^11^ These criteria are hypertension (high blood pressure, ≥130/85 mmHg), hyperglycemia (high fasting glucose, ≥100 mg/dL), hypertriglyceridemia (≥150 mg/dL [1.695 mmol/L]), low HDL-C (<40 mg/dL [male] and <50 mg/dL [female]), and central obesity (waist circumference, ≥102 cm [male] and ≥88 cm [female]).

### Ethical Considerations

This study was approved by the Biomedical Ethics Research Committee of KAUH. Informed consent was obtained from the patients. All patients were assured of the anonymity and confidentiality of data.

### Statistical Analysis

Statistical analysis was performed using IBM SPSS Statistics version 20.0. Descriptive statistics, such as frequency, percentage, mean, and standard deviation (SD), were used to describe categorical data. A p-value of <0.05 was regarded as statistically significant. Sensitivity and specificity analyses were performed. Kappa (*κ*) statistics were also performed to describe the agreement between the WHO and ATP III criteria. The relationship between the number of MetS components and T2DM complications was determined using the contingency coefficient.

## RESULTS

This study included 155 diabetes patients. The mean age was 55.6 (SD, 10.9) years. There were more females than males (n = 104, 67.1%) (Table-I). Their mean weight and height were 79.2 (SD, 18.1) kg and 161.2 (SD, 9.2) cm, respectively. Their mean BMI was 30.56 (7.1) kg/m^2^. Most patients were obese (45.8%), and the rest were overweight (37.4%), normal (15.5%), and underweight (1.3%).

**Table I:** Sociodemographic and anthropometric characteristics of patients.

Characteristics	Frequency (%) or mean ± SD
*Sociodemographic*	
Age (years)	55.6 ± 10.9
*Sex*	
Male	51 (32.9%)
Female	104 (67.1%)
*Anthropometric*	
Waist circumference (cm)	
≥102 (men) and ≥88 (women)	121 (78.1%)
<102 (men) and <88 (women)	34 (21.9%)
BMI (kg/m2)	30.6 ± 7.1
Underweight	2 (1.3%)
Normal	24 (15.5%)
Overweight	58 (37.4%)
Obese	71 (45.8%)

SD: standard deviation; BMI: body mass index.

The diabetes duration varied, with most patients having the disease for >10 years (n = 65, 41.9%). Additionally, more than half of the patients were hypertensive (n = 109, 70.3%). As depicted in Table-II, more than half were smokers, and less than one-tenth reported previous smoking. The most frequent diabetic complications included retinopathy (36.1%), neuropathy (29.7%), nephropathy (21.3%), and IHD (14.2%).

**Table II:** Clinical and biochemical characteristics of patients.

Biochemical and clinical characteristics	Frequency (%)
*Smoking*	
Smoker	11 (7.1%)
Nonsmoker	132 (85.2%)
Previous smoker	12 (7.7%)
*Blood pressure (mmHg)*	
≥130/85	109 (70.3%)
<130/85	46 (29.7%)
*Fasting blood sugar*	
≥100 mg/dL (5.6 mmol/L)	152 (98.1%)
<100 mg/dL (5.6 mmol/L)	3 (1.9%)
*High-density lipoprotein*	
<40 mg/dL (men) or <50 mg/dL (women)	113 (72.9%)
≥40 mg/dL (men) or ≥50 mg/dL (women)	42 (27.1%)
*Triglycerides*	
≥150 mg/dL	97 (62.6%)
<150 mg/dL	58 (37.4%)
*DM duration*	
<1	11 (7.1%)
1-5	38 (24.5%)
6-10	41 (26.5%)
>10	65 (41.9%)
*Retinopathy*	
Negative	69 (44.5%)
Positive	56 (36.1%)
No assessment	30 (19.4%)
*Angiopathy (PVD)*	
Negative	141 (91%)
Positive	14 (9.0%)
*Ischemic heart disease*	
Negative	133 (85.8%)
Positive	22 (14.2%)
*Ischemic stroke*	
Negative	148 (95.5%)
Positive	7 (4.5%)
*Nephropathy*	
Negative	122 (78.7%)
Positive	33 (21.3%)
*Neuropathy*	
Negative	109 (70.3%)
Positive	46 (29.7%)

DM: diabetes mellitus; PVD: peripheral venous disease.

**Fig. 1:**
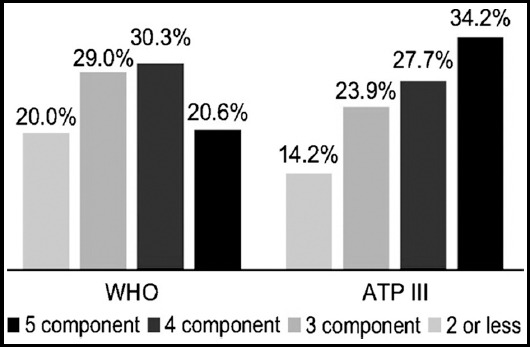
Frequency of metabolic syndrome components according to the World Health Organization (WHO) and Adult Treatment Panel (ATP) III criteria.

The prevalence of various MetS components according to the WHO and ATP III criteria are shown in Fig-1. According to the WHO criteria, 45 (29.0%), 47 (30.3%), and 32 (20.6%) patients had three, four, and five MetS components, respectively, while 31 (20.0%) patients had two or fewer MetS components. According to the ATP III criteria, 37 (23.9%), 43 (27.7%), and 53 (34.2%) patients had three, four, and five MetS components, respectively, while 22 (14.2%) patients had two or fewer MetS components.

**Fig. 2:**
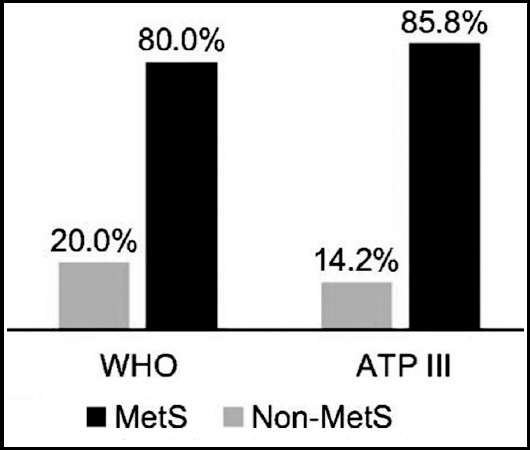
Prevalence of metabolic syndrome (MetS) according to the World Health Organization (WHO) and Adult Treatment Panel (ATP) III criteria.

The overall prevalence of MetS components (three or more components) was 80% according to the WHO criteria and 85.8% according to the ATP III criteria (Fig. 2).

The sensitivity and specificity of diagnosing MetS using the WHO versus ATP III criteria were 92.5% and 95.5%, respectively. According to the κ: statistics (κ: = 0.751, p < 0.001), the agreement between both criteria was good.

However, we found a weak positive association between the number of MetS components according to the WHO criteria and retinopathy (r = 0.270), angiopathy (r = 0.186), IHD (r = 0.126), ischemic stroke (r = 0.146), nephropathy (r = 0.09), and neuropathy (r = 0.247). Generally, there was weak positive association between the number of MetS components according to the WHO criteria and the number of diabetic complications (r = 0.317). Results also indicated a weak positive association between the number of MetS components according to the ATP III criteria and retinopathy (r = 0.309), angiopathy (r = 0.155), IHD (r = 0.059), ischemic stroke (r = 0.107), nephropathy (r = 0.123), and neuropathy (r = 0.274). In general, a weak positive association existed between the number of MetS components according to the ATP III criteria and the number of diabetic complications (r = 0.318).

## DISCUSSION

Our analysis demonstrated a high MetS prevalence among T2DM patients, irrespective of the diagnostic criteria used. This finding is consistent with those of other studies that reported prevalence rates between 58.0% and 95.8%.^2^,^7^,^12^,^13^ The high prevalence rate among our patients was expected because diabetes mellitus itself constitutes a major cardiovascular risk factor. Additionally, a relatively large proportion of our patients had a long-term disease for >10 years and were, consequently, more likely to have hyperglycemia-related complications and suboptimal glycemic control. Another obvious factor is age, which has been shown in multiple studies to be associated with MetS.^7^,^12^,^13^ Such studies have also reported that MetS prevalence increases with age;^7^,^13^,^14^ however, the peak age varies, with one study reporting a peak age of 50-70 years in men and 60-80 years in women.^15^ In another study conducted in South Africa, the peak age of MetS was 45-54 years in men and ≥65 years in women.^16^ While the average age of our patients was 55.6 (SD, 10.9) years, we have not determined the frequency of MetS by age and sex.

Similar to our report, other investigators found a higher MetS prevalence when using the ATP III criteria than when using the WHO criteria.^13^ Furthermore, the proportion of patients in our study who were identified as having different MetS components varied with the definitions used, indicating notable differences between the WHO and ATP III definitions for MetS. However, we found a good degree of agreement between both criteria because their sensitivity and specificity were 92.5% and 95.5%, respectively. The agreement of these criteria as shown by the ***κ*** statistics was 0.751 (p < 0.001). Previous studies also reported a good agreement between the WHO and ATP criteria,^7^,^17^,^18^ with a ***κ*** statistic of 0.56 in one report.^17^ In another hospital-based study that included 373 known cases of T2DM,^18^ investigators found good agreement between the WHO and ATP III criteria (***κ*** = 0.366; P < 0.001).

Although both the WHO and ATP III definitions have many notable differences, a high level of overlap is to be expected given that four out of five criteria are similar in both definitions. All our patients had diabetes and, therefore, met one of the criteria in both definitions. Subtle differences between these criteria exist in the threshold values of other MetS components (HDL, triglyceride, FBS, and blood pressure measurements), except for the cutoff values for obesity. Nevertheless, both sets of criteria allow for the inclusion of patients treated for hypertriglyceridemia and those with low HDL levels, high blood pressure, or diabetes. Consequently, the prevalence rates and agreement levels between both criteria are nearly similar for the diagnosis of MetS.

We found a weak positive association between the number of MetS components and the number of T2DM complications. Notably, most of our patients had long-standing diabetes, and more than half had hypertension. Previous studies suggested that certain metabolic risk factors co-occur, thereby increasing the risk of cardiovascular complications.^19^ Additionally, insulin resistance, suggested to be the underlying cause of MetS and one of its components as per the WHO criteria, is a potential factor that increases the risk of diabetes-associated complications.^20^-^22^ While few studies have explored the risk of CVD in diabetes patients with MetS,^23^ current data suggest a relationship between MetS and cardiovascular mortality among diabetes patients.^24^

### Limitations of the study

Our data should be interpreted within the context of the limitations of this study. First, we cannot determine which criterion had better predictive power in diagnosing MetS due to our study design. Second, our findings cannot be generalized to the population of Jeddah because this was a single-center, hospital-based study with a relatively small sample size. Third, the use of BMI as a measure of obesity is limited because disparities in fat and lean tissue have been reported across races.^25^ Thus, the BMI values in our sample may be erroneous because the sample comprised different ethnicities. Furthermore, the percentage of body fat for a specific BMI value varies with age.^25^ Despite these shortcomings, our findings could serve as preliminary data for a larger study on T2DM patients in Saudi Arabia.

## CONCLUSIONS

Our analyses revealed a high prevalence of MetS among T2DM patients regardless of the diagnostic criteria used. While a good agreement was found between the WHO and ATP III criteria, it is possible that some cases of MetS were missed by the WHO criteria. Given the high prevalence of cardiometabolic abnormalities among T2DM patients, it is imperative that clinicians identify MetS in this patient population and educate them on the importance of adherence to treatment. Health education should focus not only on treatment but also on healthy lifestyle changes.
